# Unique features of dyslipidemia in women across a lifetime and a tailored approach to management

**DOI:** 10.1016/j.ajpc.2024.100666

**Published:** 2024-04-05

**Authors:** Neeja Patel, Nikita Mittal, Michael J. Wilkinson, Pam R. Taub

**Affiliations:** a University of California, Los Angeles, United States; b University of California, San Diego, United States

**Keywords:** Dyslipidemia, Cardiovascular disease, Prevention, Lipoprotein(a), Menopause, Bempedoic acid

## Abstract

**Purpose of Review:**

Cardiovascular disease is a leading cause of death worldwide. Dyslipidemia is a critical modifiable risk factor for the prevention of cardiovascular disease. Dyslipidemia affects a large population of women and is especially pervasive within racial/ethnic minorities.

**Recent Findings:**

Dyslipidemia in pregnancy leads to worse outcomes for patients and creates increased cardiovascular risk for women at an older age. However, women remain underscreened and undertreated compared to men. Females also comprise a small portion of clinical trial participants for lipid lowering agents with increased disease prevalence compared to trial representation. However, recent lipid trials have shown different efficacies of therapies such as ezetimibe, inclisiran, and bempedoic acid with a greater relative benefit for women.

**Summary:**

Pathophysiology of dyslipidemia varies between men and women and across a woman's lifetime. While increased lipid levels or lipid imbalances are more common in postmenopausal women over age 50, conditions such as PCOS and FH produce higher cardiovascular risk for young women.

Best practices for management of women with dyslipidemia include early screening with lifestyle intervention and pharmacotherapy with statin and non-statin agents to achieve guideline directed LDL-C thresholds.

## 1 Introduction

Cardiovascular disease is a leading cause of mortality and accounts for 31 % of all deaths worldwide [1]. While cardiovascular disease can be mitigated by addressing modifiable risk factors, optimal methods for detecting and controlling these risk factors continue to evolve. Dyslipidemia, which encompasses high levels of low density lipoprotein (LDL-C), triglycerides and low levels of HDL is a critical modifiable risk factor in prevention of cardiovascular disease (CVD).

Over the past several years, the incidence and prevalence of any form of dyslipidemia in women has increased. According to the National Health and Nutrition Examination survey (NHANES) data between 2015 and 2018, 52.3 million women, (40.4 % of women) have a total cholesterol level >200 mg/dL [2]. 15.8 million women (12.1 %) have a total cholesterol >240 mg/dL and 10.3 million women (8.5 %) have a HDL-C < 40 mg/dL [2]. Several factors including age, gender, and ethnic/racial differences affect the population distribution of dyslipidemia.

Data suggests that total cholesterol levels are similar for men and women under the age of 35 years old. On average, women in this age group have lower rates of elevated total cholesterol compared to their male counterparts [3]. In men and women over the age of 50, the opposite trend was noted; women had higher rates of elevated total cholesterol and LDL-C compared to men [3]. Males and females share other similar cardiovascular risk factors. However, these risk factors also have differential effects in each biological sex [4]. For instance, metabolic syndrome in females has been identified as the most important risk factor for developing ischemic heart disease at a young age [5]. Women who smoke are more likely to develop coronary ischemia, hypertension and dyslipidemia at an older age compared to men [6], [7], [8].

The prevalence of any type of dyslipidemia (high LDL-C, low HDL-C, elevated triglycerides, or a combination) also remains stratified by racial and ethnic differences. Although minority groups make up 36 % of the US population with expectations to reach 53 % by 2050, many clinical trials with agents for treatment of dyslipidemia fail to include adequate data from these populations [9]. A three-year cross-sectional study observed female patients over age 35 from minority populations with at least one primary care visit between 2008 and 2011 to assess if minority groups were more likely to have high triglyceride/low HDL cholesterol dyslipidemia [10]. The minority groups included Filipino, Chinese, Korean, Japanese, Asian Indian, Mexican, and African American populations. Results from this study found that Filipino and Mexican women have the overall highest prevalence of dyslipidemia. Asian Indian (54.9 %) and Mexican (50.9 %) women notably have lower concentrations of HDL-C. Mexican women (45.4 %) and nearly every Asian subgroup (except Korean women) had increased prevalence of high triglycerides compared to Non-Hispanic White women (27.6 %) [10]. Black women (18.2 %) had the lowest representation within the study but had an increased proportion of dyslipidemia compared to the overall study population. Overall, Asian, Indian, Filipino, and Vietnamese women had higher risk of possessing all three dyslipidemia subtypes: high triglycerides, low HDL-C, and high LDL-C. Mexican women and every female Asian subgroup (except Japanese women) had increased risk of having combined dyslipidemia characterized by high triglycerides and low HDL-C [10].

Lipoprotein(a) (Lp(a)), an atherogenic and proinflammatory lipoprotein is an inherited and likely causative risk factor for atherosclerosis including myocardial infarction and stroke [11]. Lp(a) elevation is highly prevalent, with Lp(a) noted to be elevated in about 1.43 billion people globally [12,13]. While prevalence of elevated Lp(a) is roughly equally distributed between men and women, it varies by race/ethnicity [11]. Black individuals, both Africans and African Americans, have 2 to 3 times higher levels of Lp(a) when compared to White individuals [12]. Latin Americans have lower levels than White individuals, and Chinese individuals have lower levels than individuals of Indian origin [12].

## 2 Lipid profiles in women by age

The pathophysiology of dyslipidemia in women not only varies from pathology in men, but also varies across a woman's lifetime. There are changes in lipid levels throughout the menstrual cycle during reproductive years, during pregnancy, and post menopause.

### 2.1 The menstrual cycle

Variations in lipid concentrations occur throughout the menstrual cycle in reproductive age women, which suggests some component of hormonal regulation. Over the course of a menstrual cycle, total cholesterol and LDL-C drop in the luteal phase after the follicular phase. Directly after menses, total cholesterol and LDL-C levels rapidly increase and peak in the follicular phase, followed by a decline during the luteal phase [14]. Peak levels of total cholesterol and LDL-C during the follicular phase immediately precede the peak of estrogen, while falling cholesterol and LDL-C correspond with rising progesterone at the end of the cycle [14]. HDL-C levels peak around ovulation, correlating with high levels of estrogen. Therefore, lipid profiles measured during different points of the menstrual cycle may have some variation. Additionally, several studies including a large metanalysis of 82 trials have shown that total cholesterol levels and triglycerides are higher on average in women using oral contraceptives compared to women not taking oral contraceptives with variable effects on LDL-C [15], [16], [17].

### 2.2 Pregnancy

Pregnancy also represents a unique state characterized by increased lipid levels and increased insulin resistance [18,19]. Rises in estrogen and progesterone during the first trimester stimulate pancreatic beta cell hyperplasia with increased insulin secretion and subsequent insulin resistance [20]. Lipid synthesis and storage also increases to prepare for future fetal needs [21,22]. Changes in hepatic lipid synthesis and storage can be seen as early as 10 weeks gestation with hypertrophy of maternal adipocytes [23]. Later in pregnancy, high estrogen levels in the third trimester stimulate lipogenesis and VLDL production by the liver. Estrogen also mediates decreased clearance of lipoproteins by hepatic lipase [20]. Therefore, hormonal changes in pregnancy lead to both increased production of lipids as well as decreased clearance [24]. As a result, measured plasma cholesterol levels are 50 % higher than seen routinely prior to pregnancy and triglyceride levels are roughly doubled [25].

Numerous studies have shown that pregnant women with elevated LDL cholesterol have increased risk of gestational diabetes, preeclampsia, risk of a Cesarean delivery and preterm delivery [22,26,27]. This increased morbidity and mortality also confers a 1.8 to 4 fold greater cardiovascular risk for these mothers later in life [25,28]. Because pregnancy represents a distinctive lipid state with higher risk of complications for both mother and child, the ideal time to screen for dyslipidemia is prior to conception [29]. Guidelines from the American Heart Association and American College of Cardiology recommend a baseline lipid profile in early adulthood [30]. However, current practice demonstrates extremely low rates of early screening for lipid abnormalities: eight out of ten women of childbearing age have never had cholesterol levels checked [31]. The National Lipid Association recommends that if lipid values have not been obtained prior to pregnancy, providers should obtain lipid values at the first obstetric visit [29]. While treatment of dyslipidemia in pregnancy requires a personalized approach, providers should consider a baseline lipid profile in young women of reproductive age to assess risk prior to pregnancy.

After delivery, women are thought to maintain elevated triglycerides and total cholesterol in anticipation of lactation, which serves as a physiologic mechanism for excretion of cholesterol and triglycerides. Multiple studies have shown increased HDL-C in women who breastfed for longer, but the data regarding LDL-C, total cholesterol and triglycerides has no consistent evidence correlating with duration of breastfeeding [32,33]. While serum lipids remain elevated post pregnancy while breastfeeding, several studies demonstrate later in life cardiovascular benefit from breastfeeding [34].

### 2.3 Menopause

Cardiovascular risk for women changes significantly after age 50, aligned with the onset of menopause in many women [35]. Women's total lipid levels are typically lower than men's until age 50, after which total cholesterol becomes higher in women compared to men. Changes in lipid levels after menopause are thought to be mediated by the loss of estrogen. Metabolic changes that occur with menopause include increased visceral fat with increased adipose deposition in the abdominal cavity, increased triglycerides, LDL-C, and increased lipoprotein(a) [35]. The amount of HDL-C decreases [35]. Post-menopausal women also see greater insulin resistance and endothelial dysfunction with higher rates of hypertension and increased sympathetic tone [36]. Additionally, menopause is associated with changes in drug metabolism which can include decreased clearance of toxins and lower rates of drug metabolism [37]. Because the hormonal and atherogenic changes in older women confer increased cardiovascular risk, more aggressive treatment of hyperlipidemia should be considered. The most recent ACC/AHA guidelines do recognize menopause and pregnancy complications as a risk factor for the development of cardiovascular disease. Studies have considered the role of hormone replacement therapy in improving cardiovascular outcomes [38]. While the loss of estrogen establishes increased cardiovascular risk, multiple studies demonstrate little or unclear benefit of hormone replacement therapy for cardiovascular protection [39], [40], [41]. For some women with elevated lipoprotein(a), aspirin has been shown to be effective for primary prevention [42]. In a retrospective study utilizing data from 12,815 patients (54 % women), 406 patients were found to have a specific genotype associated with increased plasma Lp(a) levels. Individuals with the high risk variant associated with increased Lp(a) experienced risk attenuation with use of aspirin [42]. Major adverse cardiac events (MACE) reduced by 11.4 per 1000 person-years compared to 1.7 in all populations with use of aspirin (*p* < 0.008) [42]. However, limitations of this study include confining enrollment to women of European ancestry and unclear applicability to the general population.

Other considerations for cardiovascular risk in women include conditions like polycystic ovarian syndrome (PCOS) and familial hypercholesterolemia (FH) as well as modifiable risk factors such as tobacco use. PCOS, characterized by hormonal imbalance leading to increased androgens, is associated with increased obesity, insulin resistance, hypertension, and hyperlipidemia [43]. A meta analysis demonstrated cardiovascular risk in women with PCOS to be twice as high as the general population [44,45]. Thoughtful screening and consideration of early lipid management with both lifestyle and pharmacologic strategies in this population is necessary to mitigate risk [44].

Patients with familial hypercholesterolemia (FH) also require careful management of cardiovascular health. Approximately one in 250 individuals are thought to have heterozygous FH, although prevalence is likely underestimated due to low screening rates [46,47]. Inherited in an autosomal dominant pattern, individuals with this condition have a 20-fold increased risk of atherosclerotic cardiovascular disease (ASCVD) [48]. Heterozygous FH can be diagnosed as early as childhood with a screening lipid panel, but may go undiagnosed and in some cases manifest in 20–30-year-old females as complications of cardiovascular disease [46]. While cardiovascular disease typically occurs in women at a later onset than men, women with heterozygous FH have the same early age of disease onset as men [49]. Homozygous FH is much rarer with increased disease severity and earlier age of onset of atherosclerotic cardiovascular disease (ASCVD). Because 30 % of untreated women with either form of FH will have a myocardial infarction before age 60, it is essential to screen early for this condition and pursue aggressive treatment [49]. It is vital to also address modifiable risk factors such as smoking, diet, and exercise whenever possible in all patients to decrease overall risk. Lipid management for women with FH during pregnancy should involve consultation with a lipid specialist, as the risks/benefits of pharmacotherapy and in select cases, lipoprotein apheresis, must be carefully weighed.

## 3 Undertreatment of dyslipidemia in women

Women with any form of dyslipidemia are underscreened and undertreated compared to their male counterparts [50]. One retrospective study of 3793 patients estimates the proportion of women with Type 2 Diabetes receiving lipid therapy for high LDL-C levels is 10 % less than men (*p* < 0.001) [51]. Also, fewer women attained LDL-C levels at goal with lipid lowering therapy with a 33.5 % relative difference in LDL-C goal attainment rates by gender [51]. Subsequently, women experience greater disparities in outcomes, especially Black women [52,53]. As previously discussed, eight out of ten women of childbearing age have never had a lipid assessment [31]. For younger female patients with undiagnosed FH, this represents a missed opportunity for early intervention. Individuals more likely to be offered lipid screening are older, White, and with a prior history of coronary artery disease, hypertension or diabetes [52,54]. Black American and Mexican American women are screened at a lower proportion than White women, lower than men overall and are less likely to be offered lipid lowering therapy or intensification of therapy [54,55].

The Patient and Provider Assessment of Lipid Management (PALM) dataset found that women, especially younger women, are less likely to be prescribed a statin than men (67.0 % versus 78.4 %; *p* < 0.001). Women were more likely to report having never been offered statin therapy (18.6 % versus 13.5 %; *p* < 0.001) [56,57]. Not only are women less likely to be offered statins, but for patients already taking statins, appropriate intensification of lipid lowering therapy was more often offered to male patients, those with high household income, private insurance, and who were White compared to female patients, lower income, underinsured, and of non-White race/ethnicity [55]. Achievement of LDL-C < 70 mg/dL through intensification of lipid lowering therapy occurred in 32.4 % of men compared to 23.2 % women (*p* < 0.001) [55]. Women are also less likely to be prescribed aspirin (OR 0.65, 95 % CI 0.58–0.72) [58]. In addition to decreased access to preventive medications, women were more likely to decline statin therapy and more likely to perceive it as unsafe [56]. Studies have shown that women are more likely to experience statin associated muscle symptoms (SAMS) and therefore are more likely to discontinue therapy [56,59]. As a result, women may experience increased morbidity and mortality due to multiple factors: discontinuation of therapy from increased medication side-effects, decreased screening and decreased access.

This bias in treatment for lipid lowering therapies also occurs in comorbid conditions such as hypertension and diabetes. Women diagnosed with hypertension face a higher population-adjusted cardiovascular mortality compared to men and are less likely to be treated to guideline-directed blood pressure goals [60]. Women with diabetes are also less frequently prescribed evidence-based therapies by their healthcare providers. Studies have also demonstrated women who are not prescribed aggressive pharmacologic therapy for diabetes and hypertension have increased risk for major cardiac adverse events, acute coronary syndrome, and an increased ratio of ED visits per year compared to men [58]. Women with known ASCVD also experience disparities in secondary prevention. Following a myocardial infarction, younger men are more likely to begin appropriate treatment compared to women, including aspirin, statins, and participation in cardiac rehabilitation programs [61,62].

Part of the difference in provider prescription patterns can be attributed to major guidelines. Mainstays in risk stratification include an ASCVD risk calculator (Pooled Cohort Equation) which estimates 10 year risk of MI or stroke. This often leads to deferral of pharmacotherapy in women with elevated LDL-C levels since their 10 year risk may be low. Despite relatively low ten year risk, lifetime risk remains high and patients will benefit from earlier treatment of cardiovascular risk factors, including elevated LDL-C. Additionally, data have suggested that patients with elevated LDL-C but normal to high HDL-C often experience therapeutic inertia with delayed initiation of treatment for elevated LDL-C (*p* < 0.001) [63]. Elevated HDL-C should not discourage use of lipid lowering pharmacotherapy in patients with an indication for treatment. Instead, decisions around treatment should consider guideline based estimation of 10 year and lifetime ASCVD risk and the presence of risk enhancing factors and degree of LDL-C elevation. It is essential that women are offered equal, early access to treatment with alternatives if side-effects occur.

## 4 Underrepresentation of women in clinical trials of lipid-lowering therapies

Historically, far fewer women have been included in lipid medication trials compared to men. This may explain why women are less able to tolerate side effects of lipid lowering medications–they represent a smaller proportion of trial participants [64]. It has been well documented that women are less likely to tolerate statins and more likely to discontinue therapy due to adverse effects. Between 1990–2018, overall enrollment of women in lipid lowering randomized control drug trials averaged 29 % Table 1 [65]. Major modern trials that affect guidelines such as the REDUCE-IT trial, FOURIER and IMPROVE-IT included 29 %, 25 %, and 24 % of women participants respectively Table 1 [66], [67], [68]. Part of the skewed gender ratio of trial participants could be attributed to the fact that women often develop cardiovascular disease at a later age than men. Additional reasons also include requirements per protocol to exclude women of childbearing age as well as the inaccessibility of childcare to promote greater enrollment of women. However even accounting for this difference, women are greatly underrepresented in lipid trials compared to disease prevalence of dyslipidemia.

**Table 1:** Percentage of women enrolled in modern lipid lowering trials.

Trial Name and Lipid Lowering Agent	Percentage of Women Participants (%)	Year of Trial Data Publication
4S [76] (simvastatin)	18.6 %	1994
JUPITER [77] (rosuvastatin for patients with elevated high sensitivity CRP)	38.2 %	2008
IMPROVE-IT [78] (ezetimibe)	24 %	2015
HOPE-3 [79] (rosuvastatin in intermediate risk patients without CVD)	46.2 %	2016
FOURIER [80] (evolocumab)	25 %	2017
ODYSSEY Outcomes [81] (alirocumab)	25.2 %	2018
REDUCE-IT [82] (icosapent ethyl)	29 %	2019
ORION-9,10,11 pooled [83] (inclisiran)	32.5 %	2021
CLEAR Outcomes [84] (bempedoic acid)	48.5 %	2023

Underrepresentation of women in randomized control trials is paralleled by a lack of women in lipid lowering trial leadership. A recent analysis of cardiovascular trials in the last 5 years found only 10.1 % of clinical trial committee members were women, and only 17 % of first and senior authors were women [69,70]. Several new studies have demonstrated direct correlation between increased female investigators/authors and a greater proportion of women enrolled in trials with overall more diverse trial recruitment [70]. For example, a recent study showed that increased representation of women authors was independently associated with higher enrollment of women in heart failure trials [71]. Increased recruitment of diverse patient populations is crucial for more ethical patient care as well as further scientific inquiry.

It is essential to include a greater proportion of women and racially/ethnically diverse populations in randomized control trials to better understand medication efficacy and safety [70]. Sub analyses show different efficacies of lipid lowering medications in women compared to men. One such difference includes differences in drug metabolism between men and women. On average, women have higher CYP3A4 activity leading to increased drug metabolism and hepatic clearance [72]. Since the pathophysiology of dyslipidemia in women is unique and more hormonally driven than in men, greater female enrollment in lipid trials will lead to improved insights on cardiovascular prevention in women. For example, the IMPROVE-IT trial investigated the effect of ezetimibe, and only 24 % of trial participants were women [66]. Ezetimibe was found to reduce LDL by roughly the same amount in men and women but lowered major cardiac adverse events (MACE) by 12 % in women compared to 5 % in men [66]. Trials for inclisiran, a small interfering RNA that inhibits PCSK9 to lower LDL-C, demonstrated greater reduction in LDL-C in women compared to men (32.5 % female participants) [73]. Data showed the placebo-corrected mean absolute reduction in LDL-C with inclisiran at day 510 for women was 62.6 mg/dL vs 54.0 mg/dL in men with women having higher LDL-C at baseline, though percent LDL-C reduction was similar between men and women (*p* < 0.05) [73].

The most recent cardiovascular outcome trial focused on LDL-C lowering was the CLEAR Outcomes trial. This trial enrolled 13,970 patients (48.5 % women trial participants) with high CVD risk and statin intolerance to investigate the effect of bempedoic acid. An ATP citrate lyase inhibitor, bempedoic acid decreased risk of major cardiac events for males and females, but notably demonstrated greater benefit for women. A post hoc analysis of 4 studies for bempedoic acid demonstrated that a greater ratio of women experienced LDL-C reduction by 30 % compared to men (OR 1.643, *p* < 0.0096) [74,75]. With several recent trials highlighting the potential for different efficacy of lipid-lowering therapies in women compared to men, practical steps should be undertaken to develop new strategies to achieve optimal recruitment between males and females.

## 5 Dyslipidemia management in women

**Fig. 1:**
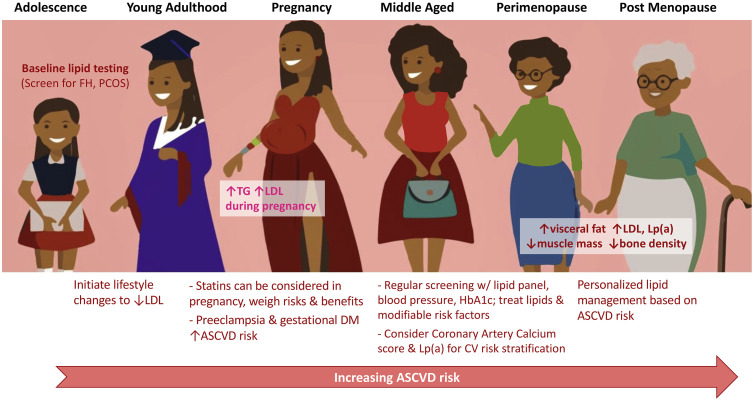
Considerations for ASCVD risk in women across a lifetime.

Current cardiovascular disease prevention guidelines recommend lifestyle modification as the initial treatment for women with dyslipidemia Fig. 1. The INTERHEART study investigated the effect of modifiable risk factors such as smoking cessation, daily fruit and vegetable consumption, and regular physical activity. The study found these interventions reduced the risk of myocardial infarction by more than 80 % [85]. Data from the World Health Organization (WHO) identified eight modifiable risk factors (alcohol consumption, smoking, hypertension, obesity, hypercholesterolemia, diabetes mellitus, low fruit and vegetable intake, and low physical activity) to account for 61 % of cardiovascular deaths and more than 75 % of causes of coronary heart disease. Although lifestyle intervention lowers risk of cardiovascular disease, the benefit disproportionately improves outcomes for men compared to women. The PREDIMED trial (Prevencion Con Delta Mediterranea) showed cardiovascular event reduction with dietary changes for the composite group, however when stratified by gender, the male subgroup had a statistically significant benefit from a Mediterranean diet while the female subgroup showed no significant endpoint difference compared to a control diet (HR 0.73, 95 % CI 0.5–1.07) [86,87].

Results from the EUROASPIRE study also note a higher prevalence of risk factors in women compared to men [85,88]. Women have higher rates of coronary microvascular dysfunction, hypercoagulability, and increased expression of concurrent metabolic syndrome [89]. Additionally, premature menopause or past pregnancy complications (preeclampsia, pregnancy induced hypertension, or gestational diabetes) increase cardiovascular risk but this may be underappreciated by many healthcare professionals [25,85,90]. Physicians should pursue pharmacologic therapy for dyslipidemia and comorbid conditions if lifestyle interventions are inadequate [90,91].

Statins have been established as the initial pharmacotherapy for patients with hypercholesterolemia to lower their cardiovascular disease risk. Several trials over the past two decades demonstrate the morbidity and mortality benefit of LDL-C reduction with statins in patients with and without vascular disease [92]. Analysis of the Justification for the use of Statins in Prevention (JUPITER) trial investigated the benefit of statin therapy in primary prevention of coronary heart disease (CHD) [93]. Results from the trial revealed a 44 % reduction in the primary composite endpoint of myocardial infarction, stroke, revascularization, unstable angina, or CHD death [93]. Recent studies show that more aggressive lipid lowering therapy with high dose statin therapy in higher risk patients provides incremental cardiovascular benefit compared to low dose or moderate dose statin therapy [86]. Incorporation of additional risk enhancing factors for initiation of statin therapy such as a coronary artery calcium (CAC) score, history of prior pregnancy complications, elevated Lp(a), inflammatory rheumatologic conditions, and comorbid fatty liver disease may provide a more detailed risk profile. Recent American College of Cardiology/American Heart Association guidelines also recommend utilization of these risk enhancing factors [94]. Integration of these factors will produce a more nuanced and tailored approach to assessing risk and treatment necessity in women.

For pregnant and breastfeeding patients with hyperlipidemia, a different approach may be necessary. Pregnant patients should first be treated with a combination of diet and exercise modification. However, lipid-lowering therapies such as bile acid sequestrants and statins could be considered. The National Lipid Association recommends colesevelam, a bile acid sequestrant, for treatment of hypercholesterolemia in pregnancy [95]. It is also considered safe while lactating because it does not enter the mother's bloodstream and therefore is not passed on to the infant. While bile acid sequestrants such as coveselam are considered safer, multiple studies found no evidence that statins cause congenital anomalies independent of concomitant medical conditions associated with their use [96]. Additionally, in 2021, the FDA removed their warning contraindicating statin use during pregnancy and reclassified the use of statins in pregnancy as acceptable in high risk patients, though may still not be recommended while breastfeeding [97]. Studies even previously hypothesized that statins may prevent the development of preeclampsia in high-risk groups, though data suggests that statin use had no effect on prevalence of preeclampsia. There is also a current, ongoing prospective study on the fetal, infant and childhood outcomes in women exposed to evolocumab during pregnancy which may provide some insight into alternative safe options during pregnancy [98]. Studies for bempedoic acid and inclisiran showed no fetal risk in animal models but no data is available in human trials [99]. Lipoprotein apheresis can be considered during pregnancy for women with FH at high risk of ASCVD events. Providers should engage patients in a risks and benefits discussion to determine the ideal method of addressing hyperlipidemia in pregnancy and while breastfeeding.

Non-statin lipid lowering agents including ezetimibe, bile acid sequestrants, PCSK9 siRNA and PCSK9 monoclonal antibodies can be considered in patients who cannot tolerate statins, prefer an alternative, or require add-on therapy to statins. Ezetimibe lowers LDL-C by roughly 13 % to 20 % and is generally well tolerated [30]. The IMPROVE-IT trial which investigated the use of ezetimibe found greater relative benefit for women, with 12 % reduction in MACE for women compared to a 5 % reduction in men [66]. PCSK9 monoclonal antibodies such as evolocumab and alirocumab may also be used for LDL-C reduction. The 2018 ACC/AHA cholesterol guidelines recommended that under certain circumstances, non-statin medications can be used in combination with statin therapy to increase the magnitude of LDL-C lowering [30]. A recent expert consensus decision pathway from the ACC published in 2022 provides guidance on use of bempedoic acid and inclisiran [94].

Successful management of patients requires that physicians universally screen and treat female patients with our current preventive therapies from an evidence based approach Fig. 1. All young female patients of reproductive age should receive a baseline lipid assessment. Engaging reproductive health providers in offering lipid assessments for pregnant and nonpregnant patients alike represents a substantial opportunity for increased screening and preventative care [100]. Physicians should also consider the possibility of FH and PCOS and address increased cardiovascular risk for these patient populations if diagnosed. Preventive medications such as aspirin and statins should be considered early with aggressive treatment in women, especially for minority women who are typically underrepresented in clinical trials.

## 6 Conclusion

Dyslipidemia affects a large population of women and is especially pervasive within racial/ethnic minorities. While it is more common in postmenopausal women over age 50, conditions such as PCOS and FH produce higher cardiovascular risk for young women. Dyslipidemia in pregnancy also leads to worse outcomes for patients and creates increased cardiovascular risk at an older age. However, women remain underscreened and undertreated compared to men. Females also comprise a small portion of clinical trial participants with increased disease prevalence compared to trial representation. However, recent lipid trials have shown different efficacies of therapies such as ezetimibe, inclisiran, and bempedoic acid with a greater relative benefit for women. Best practices for management of women with dyslipidemia include early screening with lifestyle intervention and pharmacotherapy with statin and non-statin agents to achieve guideline directed LDL thresholds.

## CRediT authorship contribution statement

**Neeja Patel:** Writing – review & editing, Writing – original draft, Project administration, Conceptualization. **Nikita Mittal:** Writing – original draft, Conceptualization. **Michael J. Wilkinson:** Writing – review & editing, Supervision, Conceptualization. **Pam R. Taub:** Writing – review & editing, Writing – original draft, Validation, Supervision, Conceptualization.

## Declaration of competing interest

The authors declare the following financial interests/personal relationships which may be considered as potential competing interests:

Pam Taub reports a relationship with Sanofi that includes: consulting or advisory. Pam Taub reports a relationship with Novo Nordisk Inc that includes: consulting or advisory. Pam Taub reports a relationship with Novartis Pharmaceuticals Corporation that includes: consulting or advisory. Pam Taub reports a relationship with Boehringer Ingelheim Corp USA that includes: consulting or advisory. Pam Taub reports a relationship with National Institutes of Health that includes: funding grants. Michael Wilkinson reports a relationship with Amarin Pharma Inc that includes: consulting or advisory. Michael Wilkinson reports a relationship with Regeneron Pharmaceuticals Inc that includes: consulting or advisory. Michael Wilkinson reports a relationship with Novartis Pharmaceuticals Corporation that includes: consulting or advisory. Michael Wilkinson reports a relationship with Regeneron Pharmaceuticals Inc that includes: speaking and lecture fees. None
